# Intratracheal dopamine attenuates pulmonary edema and improves survival after ventilator-induced lung injury in rats

**DOI:** 10.1186/cc6829

**Published:** 2008-03-10

**Authors:** Virginia Chamorro-Marín, Manuel García-Delgado, Angel Touma-Fernández, Eduardo Aguilar-Alonso, Enrique Fernández-Mondejar

**Affiliations:** 1 Unidad Experimental, Hospital Universitario Virgen de las Nieves, C/Dr. Azpitarte n°4, 18014, Granada, Spain; 2 Servicio de Cuidados Críticos y Urgencias, Hospital Universitario Virgen de las Nieves, Avda. Fuerzas Armadas n°2, 18014, Granada, Spain; 3 Servicio de Cuidados Críticos y Urgencias, Hospital Universitario Virgen de las Nieves, Avda. Fuerzas Armadas n°2, 18014, Granada, Spain

## Abstract

**Intoduction:**

Clearance of alveolar oedema depends on active transport of sodium across the alveolar-epithelial barrier. β-Adrenergic agonists increase clearance of pulmonary oedema, but it has not been established whether β-agonist stimulation achieves sufficient oedema clearance to improve survival in animals. The objective of this study was to determine whether the increased pulmonary oedema clearance produced by intratracheal dopamine improves the survival of rats after mechanical ventilation with high tidal volume (HVT).

**Methods:**

This was a randomized, controlled, experimental study. One hundred and thirty-two Wistar-Kyoto rats, weighing 250 to 300 g, were anaesthetized and cannulated via endotracheal tube. Pulmonary oedema was induced by endotracheal instillation of saline solution and mechanical ventilation with HVT. Two types of experiment were carried out. The first was an analysis of pulmonary oedema conducted in six groups of 10 rats ventilated with low (8 ml/kg) or high (25 ml/kg) tidal volume for 30 or 60 minutes with or without intratracheally instilled dopamine. At the end of the experiment the animals were exsanguinated and pulmonary oedema analysis performed. The second experiment was a survival analysis, which was conducted in two groups of 36 animals ventilated with HVT for 60 minutes with or without intratracheal dopamine; survival of the animals was monitored for up to 7 days after extubation.

**Results:**

In animals ventilated at HVT with or without intratracheal dopamine, oxygen saturation deteriorated over time and was significantly higher at 30 minutes than at 60 minutes. After 60 minutes, a lower wet weight/dry weight ratio was observed in rats ventilated with HVT and instilled with dopamine than in rats ventilated with HVT without dopamine (3.9 ± 0.27 versus 4.9 ± 0.29; *P *= 0.014). Survival was significantly (*P *= 0.013) higher in animals receiving intratracheal dopamine and ventilated with HVT, especially at 15 minutes after extubation, when 11 of the 36 animals in the HVT group had died as compared with only one out of the 36 animals in the HVT plus dopamine group.

**Conclusion:**

Intratracheal dopamine instillation increased pulmonary oedema clearance in rats ventilated with HVT, and this greater clearance was associated with improved survival.

## Introduction

Clearance of pulmonary oedema is essential for the survival of patients with acute lung injury (ALI) and acute respiratory distress syndrome (ARDS) [1,2]. Clearance of alveolar oedema depends on the active transport of sodium across alveolar epithelial type II [3-7] and probably type I cells [8], and several substances have been demonstrated to influence this mechanism. Dopamine and other β-adrenergic agonists increase pulmonary oedema clearance in different animal species [9-13] and in distinct types of lung injury [13-21]. Sustained intravenous infusion of salbutamol reduces extravascular lung water in humans with ALI or ARDS [22].

Ventilation with high tidal volume (HVT) reduces pulmonary oedema clearance in rats [23], but this effect can be reversed by intratracheal instillation of dopamine [13], resulting in improved gas exchange [24]. However, it has not been established whether dopamine achieves sufficient oedema clearance to improve survival in animals.

The objectives of the present study were twofold. First, we wished to confirm that dopamine induces enhanced clearance of pulmonary oedema in our experimental model. Second, we wished to determine whether oedema clearance from stimulation of dopaminergic receptors on alveolar epithelial cells improves the survival of rats undergoing mechanical ventilation (MV) with HVT.

## Materials and methods

### Animals

We studied 132 male Wistar-Kyoto rats weighing 250 to 300 g. A total of 60 rats were included in the pulmonary oedema study (in six groups) and 72 were included in the survival study (in two groups). All animals were purchased from the University of Granada (Spain), received food and water *ad libitum*, and were maintained on a 12:12 hours light:dark cycle. Experiments were conducted in accordance with Spanish guidelines for the ethical care of animals (Real Decreto 1201/2005).

### Preparation of instillate

Dopamine (Grifols, Barcelona, Spain) was freshly prepared before each experiment, diluting 1.5 μl in 5 ml normal saline solution.

### Surgical preparation and ventilation

Rats were anaesthetized by intraperitoneal injection of 0.8 ml/300 g of a cocktail of 5 cc ketamine (50 mg/ml) and 1 cc atropine (1 mg/ml). Additional doses were administrated when necessary to keep the animals completely anaesthetized. Only one dose was administrated to animals in the survival analysis in order to preserve their ability to breathe spontaneously after extubation. Anaesthetized animals were then placed on a servo-controlled heated table under a heating pad to maintain normal body temperature, and a tracheotomy was performed by midline incision followed by insertion of an endotracheal tube of 2.0 mm internal diameter (B/Braun, Sâo Goncalo-RJ, Brazil). Lungs were ventilated using a rodent ventilator (model 683; Harvard Apparatus, South Natick, MA, USA) connected to an oxygen supply pump to obtain an fraction of inspired oxygen of 0.5 to 0.6. Tidal volume was 8 or 25 ml/kg, respiratory rate (RR) was 40 to 50 breath/minute, and positive end-expiratory pressure of 4 cmH_2_O was applied. A catheter of 0.58 mm internal diameter (PE-50; Clay Adams, Becton Dickinson, Sparks, MD) was inserted into left carotid artery of animals in the pulmonary oedema groups to measure arterial blood gases, monitor systemic blood pressure and obtain blood samples.

### General protocol

#### Pulmonary oedema analysis groups

After surgery and a stabilization period, airway pressure (AWP) and blood pressure (BP) were measured (time = T0) in animals assigned to these groups, using calibrated pressure transducers (Transpac; Abbot, Chicago, IL, USA) connected to a monitor (Hellige Servomed, Germany, Solms). Intratracheal saline solution (2 ml/kg body weight) with or without dopamine (10^-4 ^mol/l) was then administered via endotracheal tube (PE-240; B/Braun), maintaining ventilation for 30 or 60 minutes. Ten minutes before the end of the experiment the oxygen supply pump was disconnected, continuing ventilation at fraction of inspired oxygen of 0.21, and then (at T30 or T60) AWP and BP were measured and arterial blood gases analyzed.

After each experiment, animals were exsanguinated and lungs were removed through a midline sternotomy. Both lungs were weighed and heated at 80°C for 3 days in order to determine extravascular lung water by calculating the wet weight/dry weight ratio (W/D).

#### Survival analysis groups

Rats assigned to these groups underwent MV for 60 minutes and were then extubated, closing the tracheotomy with a skin suture. Animals were then allowed to respirate spontaneously in an oxygen-rich atmosphere for 15 minutes, after which they were housed in individual cages with food and water available *ad libitum*. Survival of animals was recorded every 5 minutes for the first 40 minutes and then at 3 hours, 24 hours, 72 hours, and 7 days.

### Specific experimental protocols: pulmonary oedema groups

#### Low tidal volume for 30 minutes

In the group undergoing MV with low tidal volume (LVT) for 30 minutes (LVT-30; n = 10), MV was maintained for 30 minutes with LVT (8 ml/kg) and RR of 50 breaths/minute. At 10 minutes after starting MV, 2 ml/kg body weight saline solution was instilled intratracheally. After 30 minutes animals were killed and lungs extracted for W/D analysis.

#### High tidal volume for 30 minutes

In the group undergoing MV with HTV for 30 minutes (HVT-30; n = 10), MV was maintained for 30 minutes with HVT (25 ml/kg) and RR of 40 breaths/minute. At 10 minutes after starting MV, 2 ml/kg body weight saline solution was instilled intratracheally into air spaces. After 30 minutes the animals were killed and lungs were extracted for W/D analysis.

#### High tidal volume plus dopamine for 30 minutes

The rats underoing MV with HTV plus dopamine for 30 minutes (HVT+dopamine-30; n = 10) were subjected to the same procedure as the HVT-30 rats except that dopamine (10^-4 ^mol/l) was instilled intratracheally with the saline solution.

#### Low tidal volume for 60 minutes

In the group undergoing MV with LTV for 60 minutes (LVT-60; n = 10), MV was maintained for 60 minutes with LVT (8 ml/kg) and RR of 50 breaths/minute. At 10 minutes after starting MV, 2 ml/kg body weight saline solution was instilled intratracheally. After 60 minutes the animals were killed and lungs were extracted for W/D analysis.

#### High tidal volume for 60 minutes

In the group undergoing MV with HTV for 60 minutes (HVT-60; n = 10), MV was maintained for 60 minutes with HVT (25 ml/kg) and RR of 40 breaths/minute. At 10 minutes after starting MV, 2 ml/kg body weight saline solution was instilled intratracheally into air spaces. After 60 minutes the animals were killed and lungs extracted for W/D analysis.

#### High tidal volume plus dopamine for 60 minutes

The rats undergoing MV with HTV plus dopamine for 60 minutes (HVT+dopamine-60; n = 10) were subjected to same procedure as the HVT-60 rats except that dopamine (10^-4 ^mol/l) was instilled intratracheally with the saline solution.

### Specific experimental protocols: survival groups

#### Survival after high tidal volume

In the group in which survival was evaluated after MV with HTV (S-HVT; n = 36), MV was maintained for 60 minutes at HVT (25 ml/kg) and RR of 40 breaths/minute. At 10 minutes after starting MV, 2 ml/kg body weight of saline solution was intratracheally instilled into air spaces. After 60 minutes the animals were extubated and survival was recorded every 5 minutes for first 40 minutes and then at 3 hours, 24 hours, 72 hours, and 7 days.

#### Survival after high tidal volume plus dopamine

The rats in which survival was evaluated after HTV plus dopamine (S-HVT+dopamine; n = 36) were subjected to the same procedures as for S-HVT-60 except that dopamine (10^-4 ^mol/l) was instilled intratracheally with the saline solution.

### Statistical analysis

Data are expressed as mean values ± standard error of the mean. SPSS 13.0 for Windows (SPSS Inc., Chicago, IL, USA) was used for statistical analyses. Means of numerical variables in LVT, HVT and HVT+dopamine groups were compared using analysis of variance. Tukey's test was applied when variances of variables were normal and Dunnet's test when they were not. When the means of only two groups (HVT versus HVT+dopamine) were compared, the Student's *t*-test was applied. Finally, the Student's *t*-test for paired samples was used to study changes between times (T30 versus T60) in the same group and same variable. Survival graphs were constructed according to the Kaplan-Meier method, and the log-rank test was used to compare curves. A contingency table was used to conduct comparisons between HVT groups with and without intratracheal dopamine administration. *P *< 0.05 was regarded to represent statistical significance.

## Results

### Wet weight/dry weight ratio

**Figure 1:**
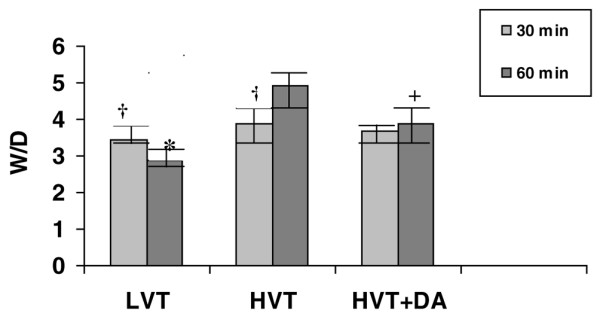
Wet eight/dry weight ratio. At the end of experimental period, wet weight/dry weight ratio (W/D) was determined in rats ventilated with low tidal volume (LVT), high tidal volume (HVT), and HTV plus 10^-4 ^mol/l dopamine (HVT+DA). Values are expressed as means ± standard error of the mean. *Statistically significant difference in the comparison of the LTV for 60 minutes group with the HTV for 60 minutes group and the HTV+DA for 60 minutes group (*P *= 0.001 and *P *= 0.007, respectively). ^+^Statistically significant difference in the comparison of the HVT for 60 minutes group with the HVT+DA for 60 minutes group (*P *= 0.014). ^†^Statistically significant differences between the LVT for 30 minutes group and the LVT for 60 minutes group, and between the HVT for 30 minutes group and the HVT for 60 minutes group (*P *= 0.005 and *P *= 0.022).

W/D was similar among the three groups ventilated for 30 minutes, regardless of the tidal volume level or whether intratracheal dopamine was administered. W/D ratio was higher in rats ventilated at HVT for 60 minutes (4.9 ± 0.29 for HVT-60 and 3.9 ± 0.27 for HVT+dopamine-60) than in rats ventilated at LVT for the same period of time (2.89 ± 0.13 for LVT-60; *P *= 0.001 and *P *= 0.007 versus HVT-60 and HVT+dopamine-60, respectively; Figure 1). W/D ratio was lower in rats ventilated at HVT and instilled with dopamine than in rats ventilated at HVT without dopamine (3.9 ± 0.27 for HVT+dopamine-60 versus 4.9 ± 0.29 for HVT-60; *P *= 0.014).

W/D ratio was higher in rats ventilated with LVT for 30 minutes than in rats ventilated for 60 minutes (3.46 ± 0.04 for LVT-30 versus 2.89 ± 0.13 for LVT-60; *P *= 0.005). However, in rats ventilated at HVT without dopamine, W/D ratio was lower after ventilation for 30 minutes than after ventilation for 60 min (3.89 ± 0.25 for HVT-30 versus 4.9 ± 0.29 for HVT-60; *P *= 0.022).

### Arterial oxygenation and other measurements

In groups ventilated at HVT without intratracheal dopamine, oxygen saturation deteriorated over time and was higher after 30 minutes than after 60 minutes (96.7 ± 2.3% for HVT-30 versus 86.8 ± 2.6% for HVT-60; *P *= 0.014; Table 1). In animals ventilated with HVT and receiving dopamine, oxygen saturation was again better after 30 minutes than after 60 minutes (95.4 ± 0.5% for HVT+dopamine-30 versus 91.1 ± 1.43% for HVT+dopamine-60; *P *= 0.05; Table 1). Partial carbon dixoide tension values were significantly higher after 60 minutes than after 30 minutes (*P *= 0.05) in animals receiving dopamine; pH varied as a function of changes in partial carbon dixoide tension (Table 1).

**Table 1:** Oxygenation measurements (30 and 60 minutes)

	LVT-30	LVT-60	HVT-30	HVT-60	HVT+dopamine-30	HVT+dopamine-60
SO_2 _(%)	98 ± 2.10	95 ± 1.7	96.7 ± 2.30	86.8 ± 2.6*	95.4 ± 0.5	91.1 ± 1.43*
PO_2 _(mmHg)	120 ± 7.5	117 ± 5.4	112.5 ± 10.0	81.9 ± 4.2	122 ± 3.3	98.1 ± 7.97*
PCO_2 _(mmHg)	30 ± 2.20	35 ± 3.4	28.2 ± 2.90	36.3 ± 5.5	21.4 ± 1.97	31 ± 2.00*
pH	7.41 ± 0.01	7.38 ± 2.10	7.39 ± 0.01	7.34 ± 0.02	7.49 ± 0.03^†^	7.39 ± 0.02*

Ten animals were included in each group. Values are expressed as means ± standard error of the mean. *Statistically significant differences between high tidal volume for 30 minutes (HVT-30) and HVT for 60 minutes (HVT-60) groups (*P *= 0.014), and between HVT plus dopamine for 30 minutes (HVT-dopamine-30) and HVT plus dopamine for 60 minutes (HVT+DA-60) groups (*P *= 0.05). ^†^Statistically significant difference between HVT-30 and HVT-30+DA groups (*P *= 0.023). PCO_2_, partial carbon dioxide tension; PO_2_, partial oxygen tension; SO_2_, oxygen saturation.

At the end of the experiment, the mean BP was lower in the LVT-60 group than in the LVT-30 group (*P *= 0.025). Among the groups ventilated for 60 minutes, MBP was significantly higher in the HVT+dopamine-60 group than in the LVT-60 group (*P *= 0.018; Table 2).

**Table 2:** Measurements of mean BP and AWP (30 and 60 minutes)

Time	Start of experiment	End of experiment
Mean BP (mmHg)		
LVT-30	103.8 ± 1.4	87.6 ± 4.0
LVT-60	105.3 ± 2.3	75.3 ± 5.2^‡^
HVT-30	103.8 ± 1.4	93 ± 5.1
HVT-60	105.3 ± 1	88 ± 4.6
HVT+dopamine-30	106.3 ± 1.7	92.3 ± 2.8
HVT+dopamine-60	106.5 ± 2.5	97.5 ± 3.8^†^
AWP (mmHg)		
LVT-30	14.8 ± 1.1*	16.6 ± 1.1*
LVT-60	13.9 ± 1.2*	18.3 ± 0.8*
HVT-30	30 ± 1.7	28.8 ± 1.4
HVT-60	31 ± 1.8	28 ± 1.6
HVT+dopamine-30	24.6 ± 0.6	23 ± 0.9^+^
HVT+dopamine-60	27.5 ± 1.9	26.5 ± 1.3

Ten animals were included in each group. Values are expressed as means ± standard error of the mean. *Statistically significant difference for the comparison of the LVT-30 group with HVT-30 and HVT+DA-30 groups, and of the LVT-60 group with HVT-60 and HVT+DA-60 groups (*P *= 0.001) at the beginning and end of the experiment. ^+^Statistically significant difference between HVT-30 and HVT+DA-30 groups (*P *= 0.012) at the end of the experiment. ^†^Statistically significant difference between LVT-60 and HVT+DA-60 groups (*P *= 0.018) at the end of the experiment. ^‡^Statistically significant difference between LVT-30 and LVT-60 groups (*P *= 0.025) at the end of experiment. AWP, airway pressure; BP, blood pressure; HVT-30, high tidal volume for 30 minutes; HVT-60, high tidal volume for 60 minutes; HVT+dopamine-30, high tidal volume plus dopamine for 30 minutes; HVT+dopamine-60, high tidal volume plus dopamine for 60 minutes; LVT-30, low tidal volume for 30 minutes; LVT-60, low tidal volume for 60 minutes.

A tendency was observed for the AWP to increase in groups ventilated with LVT and to decrease in groups ventilated with HTV (Table 2).

### Survival

**Figure 2:**
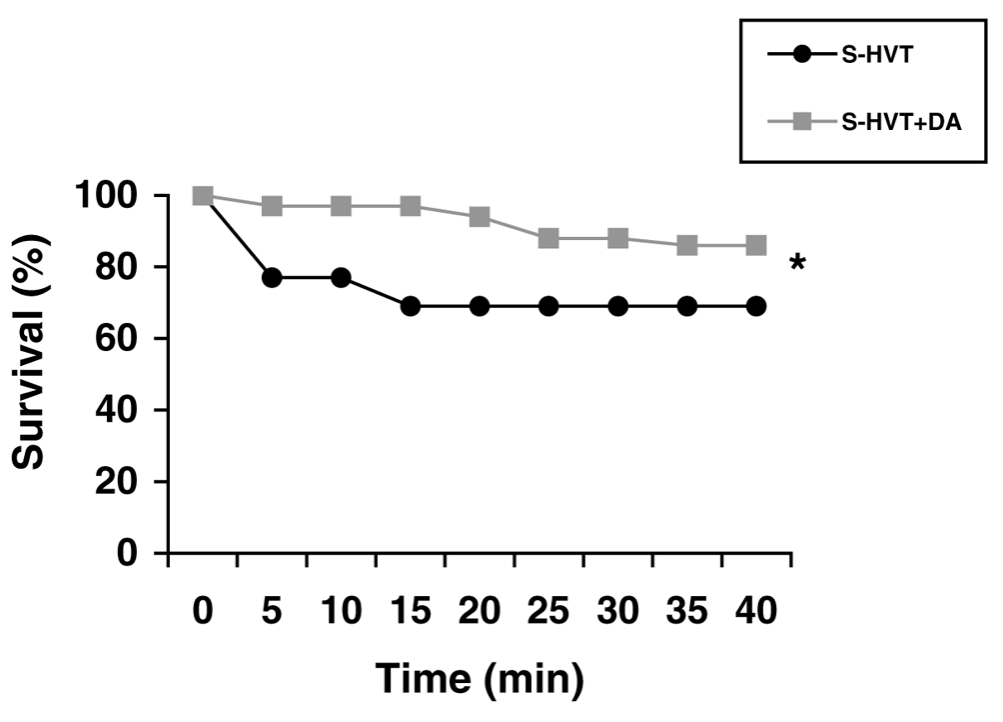
Survival. Survival was higher in the dopamine-treated group throughout the experiment. *Significant difference between groups (*P *= 0.0132).

Rats ventilated with HVT that received intratracheal dopamine exhibited significantly lower mortality compared with those not receiving this treatment (*P *= 0.013; Figure 2).

Fifteen minutes after extubation, 11 animals out of the 36 (30%) in the S-HVT group had died versus only one of the 36 (2.7%) in the S-HVT+dopamine group. Between 15 minutes and 35 minutes, four more animals in the S-HVT+dopamine group died as compared with none in the S-HVT group. From 35 minutes to the end of follow up at 7 days, no animals died in either group.

## Discussion

This study demonstrates that intratracheal instillation of dopamine reduces pulmonary oedema in rats receiving MV with HVT and is associated with a higher survival rate, probably because of improved gas exchange.

Various studies [25-29] have found that administration of dopamine or other β-adrenergic agents stimulates pulmonary oedema clearance via the Na^+^/K^+^-ATP pump. However, if it is to be regarded biologically relevant, a greater pulmonary oedema clearance must be accompanied by improvements in other physiological parameters, such as arterial blood gases. Only a few studies have addressed this issue [21,24], and these found that β-adrenergic stimulation improved gas exchange in sheep and rats with hydrostatic or smoke inhalation lung injury. The present study demonstrates that intratracheal dopamine instillation after ventilation with HVT (25 ml/kg) for 60 minutes produces a sufficiently large reduction in pulmonary oedema to improve survival.

### Pulmonary oedema development

Development of pulmonary oedema was similar among the three oedema evaluation groups after 30 minutes of ventilation but differed significantly after 60 minutes. Thus, in animals ventilated with LVT, pulmonary oedema was greater at 30 than at 60 minutes. It may be that some instilled liquid remains after 30 minutes and continues to be absorbed over the subsequent 30 minutes, reducing the amount of lung water observed at 60 minutes (Figure 1). Groups ventilated with HVT exhibited a different behaviour. Ventilation with HVT produced a significant increase in pulmonary oedema in the groups receiving saline solution alone. However, in the groups receiving intratracheal dopamine there were minimal differences between observations at 30 and 60 minutes. This may indicate that the oedema produced by ventilation with HVT was reabsorbed during the second 30 minute period.

### Gas exchange

Ventilation with HVT produced a time-dependent impairment in arterial oxygen saturation, oxygenation and pH. Values were significantly worse after 60 minutes than after 30 minutes in animals with or without intratracheal dopamine. After 60 minutes oxygen saturation was 91% in the group with dopamine versus 86% in the group without. Although the percentage difference was not statistically significant, it should be taken into account that these arterial oxygen saturation values were obtained during MV. This difference was probably greater after withdrawal of MV in groups selected for survival analysis and may have influenced the survival of these animals.

### Lung injury due to overdistension

A major challenge in this type of survival analysis is to identify a lung lesion that is sufficiently large to be detectable but sufficiently small to allow the survival of some treated groups. Ventilation with HVT produces lung injury with the formation of pulmonary oedema [14-20] and a decrease in oedema clearance [23]. The intensity of the oedema and reduction in its clearance is related to the tidal volume used and the duration of MV. Lecuona and coworkers [23] reported, in an *ex vivo *model, that a tidal volume of 40 ml/kg with 35 cmH_2_O of mean AWP produced severe pulmonary oedema and a major decrease in its clearance, whereas a tidal volume of 30 ml/kg with 20 cmH_2_O of mean AWP had no effect on pulmonary oedema or its clearance. Nin and colleagues [30] found that a tidal volume of 35 ml/kg induced moderate lung injury associated with a postextubation mortality rate of around 50%. A tidal volume of 25 ml/kg was used in the present study, which could be expected to produce moderate lung injury with oedema and a moderate effect on pulmonary oedema clearance.

It has been argued that the alveolar overdistension model using HVT is not physiological and is of little clinical relevance. Unfortunately, however, prescribed tidal volumes can be greatly exceeded in the clinical setting, for instance during emergency cardiopulmonary resuscitation or when selective intubation of the left or right bronchus remains undetected for some time, and the resulting alveolar overdistension can produce lung injury.

### Haemodynamics and airway pressures

After both 30 and 60 minutes, groups ventilated with LVT exhibited a lower AWP versus groups ventilated with HVT, whether or not they received intratracheal dopamine (Table 2). Interestingly, the AWP was higher in the HVT-30 group than in the HVT+dopamine-30 group at the end of the experiment, which cannot be accounted for by greater clearance of the pulmonary oedema because this did not significantly differ between the groups. This finding was probably influenced by the baseline AWP values, which appeared to be lower in the HVT-30 group, with a difference that was not significant at baseline but reached significance at 30 minutes when the effect of dopamine was added.

At the end of the experiment, the mean BP was lower in the LVT-60 group than in the HVT+dopamine-60 group. The intratracheal dopamine possibly had some haemodynamic effect, improving the BP. We do not know whether this finding could have been influenced by other factors that were not considered in this study. In the groups ventilated with LVT the mean BP was lower in the LVT-60 group than in the LVT-30 group at the end of the experiment. Although this difference was statistically significant, the mean BP in both groups can be considered to be within the physiological range.

### Survival

Survival was significantly superior in rats ventilated with HVT and receiving intratracheal dopamine than in those not receiving this treatment (*P *= 0.013). Thus, in the survival analysis of animals ventilated with HVT, only one out of 36 animals in the dopamine-instilled group died during the first 15 minutes, with a further four dying after this time. This may be because the oxygen-rich atmosphere in which the rats were maintained for the first 15 minutes after extubation provided sufficient support to keep alive moderately hypoxaemic animals that then died after removal of the oxygen. In contrast, 11 out of 36 of the animals that did not receive dopamine died during the first 15 minutes, which might suggest that their lung injury was too severe for them to be kept alive by the oxygen-rich atmosphere. This hypothesis is supported by observations in the animals ventilated at HVT for 60 minutes for oedema assessment (groups HVT-60 and HVT-60+dopamine); in these rats a trend toward higher arterial oxygen saturation (absolute difference 5%) was observed in animals receiving dopamine than in those not receiving this treatment. As noted above (see Gas exchange, above), although arterial oxygen saturation data were recorded at the end of the experiment, the animals were still receiving MV, and differences in oxygenation may be greater under conditions of spontaneous respiration. In reported clinical investigations, oxygenation has not consistently been found to be associated with survival, but it should be taken into account because all other support measures are maintained in the clinical setting. In contrast, in experimental studies, when no other support measures are applied, oxygenation may play a crucial role in survival. In the same way, animals receiving dopamine appear to have a higher mean BP, but we do not know whether instilled intratracheal dopamine can reach the systemic circulation and how this might affect the survival.

Few studies have analyzed the survival of rats subjected to alveolar overdistension. The higher survival rate (69%) in the present investigation than in the study conducted by Nin and coworkers [30] (50%) may be accounted for by the higher tidal volume used in the latter study (25 versus 35 l/kg).

This study was conducted in rats, and it should be taken into account that animals of such small size are known to have a much higher pulmonary oedema clearance than larger animals [10,31,32]. Therefore, these positive results require confirmation in other animal species. Nevertheless, the higher survival found in animals receiving intratracheal dopamine is consistent with a recent retrospective clinical study [33] that compared a high with a low dose of inhaled β_2_-agonist (salbutamol) in ALI patients; it found the higher dose to be associated with better outcome.

## Conclusion

In conclusion, intratracheal dopamine administration in a rat model of ventilator-induced lung injury improves clearance of pulmonary oedema, and this greater clearance is associated with improved survival.

## Key messages

• The increased clearance of pulmonary oedema resultinng from intratracheal instillation of dopamine is associated with higher survival.

## Abbreviations

ALI = acute lung injury; ARDS = acute respiratory distress syndrome; AWP = airway pressure; BP = blood pressure; HVT = high tidal volume; LTV = low tidal volume; MV = mechanical ventilation; RR = respiratory rate; W/D = wet weight/dry weight ratio.

## Competing interests

The authors declare that they have no competing interests.

## Authors' contributions

VCM conducted the experiments and co-wrote the manuscript. MGD, ÁTF and EAA all participated in the experiments. EF-M designed the study protocol and co-wrote the manuscript.
